# Transcriptome Landscape Analyses of the Regulatory Network for Zygotic Embryo Development in *Paeonia ostii*

**DOI:** 10.3390/ijms241310715

**Published:** 2023-06-27

**Authors:** Yufeng Xu, Wenqian Shang, Linda Li, Yinglong Song, Guiqing Wang, Liyun Shi, Yuxiao Shen, Yuke Sun, Songlin He, Zheng Wang

**Affiliations:** 1 College of Landscape Architecture and Art, Henan Agricultural University, Zhengzhou 450002, China; xuyufeng_2011@163.com (Y.X.); wenqianshang@henau.edu.cn (W.S.); linda318428@163.com (L.L.); edward_song1989@163.com (Y.S.); guiqingw@hotmail.com (G.W.); sisyrin@henau.edu.cn (L.S.); yxshen@henau.edu.cn (Y.S.); yukesun1@163.com (Y.S.); 2 Henan Institute of Science and Technology, Xinxiang 453000, China

**Keywords:** *Paeonia ostii*, embryo development, transcriptional regulation, gene profiling, early embryonic

## Abstract

*Paeonia ostii* is a worldwide ornamental flower and an emerging oil crop. Zyotic embryogenesis is a critical process during seed development, and it can provide a basis for improving the efficiency of somatic embryogenesis (SE). In this study, transcriptome sequencing of embryo development was performed to investigate gene expression profiling in *P. ostii* and identified Differentially expressed genes (DEGs) related to transcription factors, plant hormones, and antioxidant enzymes. The results indicated that IAA (Indole-3-acetic acid), GA (Gibberellin), BR (Brassinosteroid) and ETH (Ethylene) were beneficial to early embryonic morphogenesis, while CTK (Cytokinin) and ABA (Abscisic Acid) promoted embryo morphogenesis and maturation. The antioxidant enzymes’ activity was the highest in early embryos and an important participant in embryo formation. The high expression of the genes encoding fatty acid desaturase was beneficial to fast oil accumulation. Representative DEGs were selected and validated using qRT-PCR. Protein-protein interaction network (PPI) was predicted, and six central node proteins, including AUX1, PIN1, ARF6, LAX3, ABCB19, PIF3, and PIF4, were screened. Our results provided new insights into the formation of embryo development and even somatic embryo development in tree peonies.

## 1. Introduction

Seeds, as a unit of plant reproduction, carry genetic information to the next generation of the plant [1]. Zyotic embryogenesis is a critical process during seed development, and it can be roughly divided into three stages: histodifferentiation, embryo patterning and growth, and embryo maturation, and further divided into 6 stages: proembryo, globular, heart, torpedo, cotyledon, and maturation [2,3,4]. Seed development is one of the key and complex processes for plant growth and development and is regulated by integrated molecular regulatory networks of temporal and spatial, especially the regulation of various types of transcription factors, plant hormones, and antioxidant enzymes [1,5].

A large number of key transcription factors play an important role in plant embryogenesis, including *LEAFY COTYLEDON* (*LEC*), *AGAMOUS*-*Like15* (*AGL15*), *ABSCISIC ACID INDPENDENT3* (*ABI3*), *BABY BOOM* (*BBM*), *FUSCA3* (*FUS3*), *WUSCHEL* (*WUS*), *SOMATIC EMBRYOGENESIS RECEPTOR*-*LIKE KINASE* (*SERK*), and *CUP SHAPED COTYLEDONS* (*CUC*) [6,7]. *LEC1* and had a high expression during early embryo development in *Theobroma cacao* and *Zea mays* [8,9]. *LEC1*, *ABI3*, *FUS3*, and *LEC2* are called LAFL factors, and they are necessary and sufficient for embryo development and regulate various development processes [10,11]. *SERKs* are active in regulating embryonic morphogenesis. Pineapple *SERK1* Promoter was increasing during embryogenic acquisition [12], and *ZmSERK1* and *ZmSERK2* had high expression in maize embryogenic callus [13,14]. *TaSERK2* and *TaSERK3* were involved in auxin-specific responses, whereas *TaSERK1*, *4*, and *5* were more specific for BR-mediated regulation [15]. *AGL15* can directly target *LEAFY COTYLEDON2*, *FUSCA3*, and *ABA INSENSITIVE 3* to participate in embryonic development [16]. These genes form a complex regulatory network of embryogenesis.

Plant hormones play an important role in seed development [17,18]. IAA (Indole-3-acetic acid) is a key component in seed development; the spatial-temporal distribution of seed development is dynamically regulated by IAA biosynthesis and catabolism, transport, and signal transduction [18,19,20]. CTK (Cytokinin) occurs in the phase of rapid cell division, which is critical in establishing the final seed size. GA (Gibberellin) and IAA regulate the transport of nutrient substances during seed development. ABA (Abscisic acid) contributes to maintaining embryogenesis during the nutrient accumulation process [21]. Auxin importers AUX1/LAX play an important role in embryonic root formation. Radicle development was the disorganization of *aux1, lax1, lax2,* and *lax3* quadruple mutants [22]. Auxin response factor *ARF16* had the highest expression in early embryos of *Pinus pinaster* [23]. YUCCA flavin monooxygenases play an essential role in *Arabidopsis* embryogenesis, RNA in situ hybridization showed *YUC1* and *YUC4* were mainly expressed in globular and heart embryo stages [24]. *YUC4* is a transcriptional target of *LEC2* [25]. GA/ABA may determine the pre-embryonic or post-embryonic development of embryos [16]. *AGL15* can directly target the downstream gene gibberellin 2-oxidase (*GA2ox6*) and further regulate GA changes during embryonic development in *Arabidopsis* [26]. Auxin affects the expression level of *FUS3*, while *FUS3* negatively regulates the expression of GA biosynthetic genes *GA3ox1* and *GA3ox2* [27,28]. *FUS3* is a link between hormones during embryogenesis [27].

Research shows that enzymes, including antioxidant enzymes, that respond to oxidative stresses have been used effectively as efficient biochemical markers to study embryonic development [29]. The high content of antioxidant enzymes has a stimulatory effect on embryogenesis [30]. During embryogenesis, many enzymes (POD, SOD, CAT, and APX) actively regulate ROS and hormones to prevent cellular damage in plants [31,32,33,34,35,36]. In conclusion, plant hormones and enzyme activity play an important role in seed development.

Transcriptome sequencing can comprehensively and quickly obtain transcripts of specific organs or tissues at a certain state, and it is an important technical way to reveal the gene expression profiling of cells and tissues [37,38,39,40]. Research showed that auxin was crucial for early embryo patterning and pre-cotyledon embryonic formation, while ABA was a major regulator of embryonic maturation by transcriptome, the key genes related to embryo development were obtained and used to improve the efficiency of the somatic embryo (SE) in *Picea mongolica* [41,42]. Transcription factors, hormone signals, and transduction-related and sugar-metabolism-related genes play a crucial role in barley grain development [3,43]. In recent years, many studies have focused on the fatty acid synthesis and metabolism in tree peonies; however, there are few studies on the gene regulation of embryo development [44,45,46,47].

Tree peony, belonging to the genus *Paeonia* L. and the family Paeoniaceae, is a world-renowned perennial deciduous shrub with ornamental and medicinal values [48,49]. However, tree peony has a long generation cycle, a single reproduction method and a small reproduction coefficient, which hinders the large-scale production of their seedlings [50]. The somatic embryo regeneration pathway is an important part of the plant tissue culture and one of the most effective ways to achieve rapid reproduction of tree peonies [7,40,51]. Nevertheless, the transformation efficiency of non-embryogenic callus to embryonic callus in somatic embryogenesis is still low [52], which may be due to superficial abnormalities in the gene expression of hormones, antioxidant enzymes, and transcription factors during the transformation process in tree peony. Studies have shown that somatic embryos and zygotic embryos were similar in morphological characteristics, even at the biochemical level, especially the similarity of gene expression products and protein components [53,54]. Therefore, it is an effective way to break through the technical difficulties of tissue culture by clarifying the normal development process of seedling embryos and exploring the expression profiles of key genes such as hormones, antioxidant enzymes, and transcription factors. The molecular mechanism of zygotic embryo development can provide a theoretical and technical basis for the development of somatic embryos of tree peonies.

In this study, paraffin sections were made to observe the critical period of the embryo development of *P. ostii*. Furthermore, we divided the process into 7 stages: proembryo (PE), globular embryo (GE), heart embryo (HE), torpedo embryo (TE), cotyledon embryo (CE), not fully mature embryo (NE), and mature embryo (ME). Then we analyzed the gene expression profile during the seed development of *P. ostii* using RNA-seq, especially hormones, enzyme activity-related genes, and various transcription factors. Protein-protein interaction network (PPI) related to early embryogenesis was constructed. The spatiotemporal gene expression profile of embryo development and PPI of early embryogenesis provided an important resource for the development of zygotic embryos and even somatic embryos in the plants.

## 2. Results

### 2.1. Morphological Observation of Tree Peony Pod Development after Pollination

**Figure 1:**
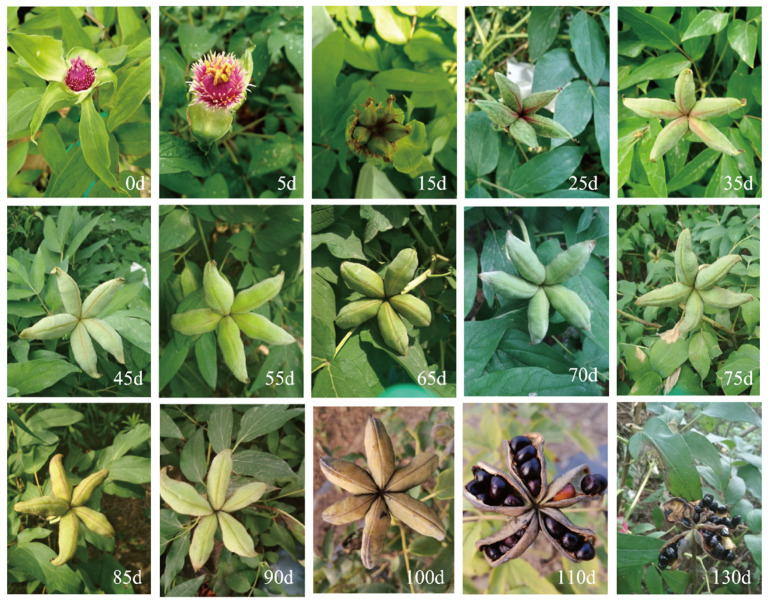
Pods and seed development state of *P. ostii*. 0 d (days), 5 d, 15 d, 25 d, 35 d, 45 d, 55 d, 65 d, 70 d, 75 d, 85 d, 90 d, 100 d, 110 d, and 130 d represent the observation time after pollination.

After the pollination (0 days) of *P. ostii* (Figure 1), the stigma was pink, and the seed pods were gathered together; the inside of the seed pods was milky white, there was no mucus in the pods, and the seeds were not visible to the naked eye. At 3–5 days, the stigma was red, with mucus and a shiny surface, and smaller seeds can be observed after peeling off; the stigma became dry and yellow-black at 15 days, and the five green pods were separated; At 25 days, the pods had a small amount of red in the slits, while whole pods became enlarged, and a lot of mucus in the pods, and the seeds were small and milky white; At 35 days, the pods turned dark green, and part of the sutures was red inside the pods. There was a large amount of mucus in the pods, the milky white seeds were obviously enlarged, and some aborted seeds turned yellow-black; for 45–85 days, the seed pods were completely dark green and became full and swollen, there was a large amount of mucus in the pods, and the seeds changed from milky white to yellow. At 90 days, the yellow pods had brown spots, and a small portion of the pods had dried out. At 110 days, the pods turned blackish-yellow, dehydrated with partial dehiscence and no mucilage in the pods, and the seeds exposed after the pods had cracked began to turn black and hard; At 130 days, the pods were dry and completely cracked, and the black seeds were exposed.

### 2.2. Anatomical Observation of Embryo Development of Tree Peony

**Figure 2:**
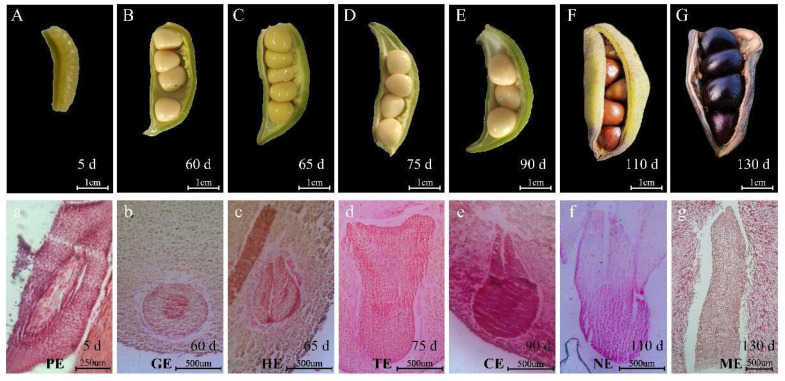
The Seed characteristics and the microscopy images of paraffin-embedded tissues of the embryo. (**A**–**G**) The seed characteristics at 5, 60, 65, 75, 90, 110, and 130 days after pollination; (**a**–**g**) The development status of the embryo at 5, 60, 65, 75, 90, 110, and 130 days after pollination was PE, GE, HE, TE, CE, NE, ME, respectively. PE, GE, HE, TE, CE, NE, and ME represent proembryo, globular embryo, heart embryo, torpedo embryo, cotyledon embryo, not fully mature embryo, and mature embryo. The scale bar is shown in the lower right corner, (**A**–**G**) Scale bar = 1 cm; (**a**) Scale bar = 250 µm; (**b**–**g**) Scale bar = 500 µm.

The paraffin section method was used to anatomically observe the development process of the embryo of *P. ostii*. After pollination, the sperm cells and oocytes combine to form a zygotic embryo, and the embryo initially develops and polarizes into a mature embryo sac (Figure 2A). Globular embryos can be observed in the ovules about 60 days after pollination (Figure 2B); when the globular embryo developed to a certain stage, two bulges were produced on both sides near the chalazal end at the same time, namely, the cotyledon primordium. The cotyledon primordium gradually increased and developed into a heart-shaped embryo (Figure 2C), and embryogenesis was a sign that organ differentiation had begun. The conversion of heart-shaped embryos into torpedo embryos can be observed in the ovules 75 days after pollination (Figure 2D). The two cotyledons of the torpedo embryo elongated rapidly, and at the same time, the embryo growth point began to differentiate and form and entered the cotyledon embryo stage (Figure 2E); the cotyledon embryo mostly appeared in the ovule about 90 days after pollination. After the cotyledon embryo was formed, the embryo body had no obvious changes in shape, but the embryo body would continue to grow. From 90 days to 130 days after pollination, the embryo increased, while the radicle, hypocotyl, and embryo growth points became increasingly obvious (Figure 2F,G). As the main location for accumulating nutrients, the cotyledons no longer showed significant changes by 130 days after pollination. At this time, the embryo matures and enters a dormant state.

On the basis of the paraffin analysis results, we divided the development into the proembryo (PE), the globular embryo (GE), the heart embryo (HE), the torpedo embryo (TE), the cotyledon embryo (CE), the not fully mature embryo (NE), and the mature embryo (ME) periods. It takes a long time to progress from PE to GE, about 55 days, which is related to the dramatic changes in the biological processes during this period. The process changes of GE-HE-TE-CE were faster and only required 30 days. After the embryo matures, the size of the embryo and seed is basically unchanged.

### 2.3. Antioxidant Enzymes Activities during the Seed Embryo Development of P. ostii

**Figure 3:**
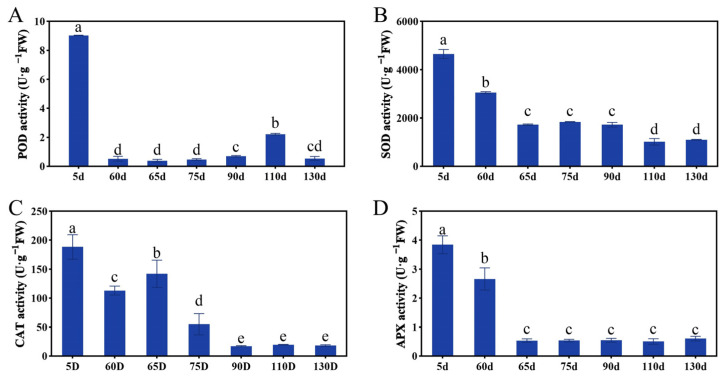
The POD (**A**), SOD (**B**), CAT (**C**), and APX (**D**) enzyme activities during the development of peony seed embryos. The letters above the bars indicates the significance among different samples. Bars represent means ± standard error (n = 3). *p* < 0.05, one-way ANOVA.

Antioxidant enzymes have been used effectively as efficient biochemical markers to study embryonic development [29]. To explore the antioxidant enzyme activities of embryo development in *P. ostii*, we examined the changes of POD, SOD, APX, and CAT activity during seed development, and the results showed that their enzyme activity was particularly active in the early stage and then downregulated (Figure 3). POD activity was high at 5 days, rapidly decreasing at 60 days and maintaining a relatively low level until embryo maturity. The activity of SOD, CAT, and APX was the highest at 5 days, decreased at 60 days, decreased to a lower level at 65 days, and maintained until embryo maturation. As shown in Figure 2, the embryo was in the proembryo period 5 days after pollination, and sperm cells and egg cells combined to form fertilized eggs and continued to divide to complete morphogenesis. It can be seen that antioxidant enzyme activity is closely related to embryo morphogenesis.

### 2.4. Global Analysis of the Time-Course Transcriptome Data in Embryo Development

To explore the molecular mechanism of embryo development of *P. ostii*, a total of 21 cDNA libraries constituting three biological repeats were constructed from seven stages of developing seeds (Figure 2). In total, approximately 79.8–91.35 M clean reads were obtained from the PE, GE, HE, TE, CE, NE, and ME samples. The average Q20 contents among the 21 samples ranged from 96.85% to 97.28%. These data indicate that the sequencing results were acceptable (Table S2).

The annotation results of GO and KOG showed that genes mined from the transcriptome were involved in a variety of biological processes (Figure S1). The species with the best match for each gene showed a 26.06% match with *Vitis vinifera* (Figure S2A). The statistical results showed that a large number of genes were highly expressed in the PE period, and various genes were up-regulated or down-regulated in each period of *P. ostii* embryo development (Figure S2B; Table S3).

### 2.5. Gene Expression during Different Embryo Development Stages

To assess the repeatability and similarity among our datasets, principal component analysis (PCA) was performed. The results showed that the three replicates of each sample were located near each other, except NE2, and NE2 was similar to the CE stage (Figure S3A). The samples were similar from the beginning of embryo development to the early stage of embryo maturity. The PE stage clustered independently, which was obviously different from other developmental stages. NM was the mature stage of seed embryo development, which was different from other stages.

Since embryo development in different stages showed distinct expression profiles, we defined the genes with FPKM < 1 in all samples and FPKM ≥ 3 in any of the PE samples as embryo-specific genes, and so on. Using this filter condition, we obtained 3578 specific expression unigenes and identified 2733 (76.3%), 88 (2.5%), 121 (3.4%), 38 (1.1%), 43 (1.2%), 173 (4.8%), and 381 (10.6%) specific expressing genes in PE, GE, HE, TE, CE, NE, and ME, respectively (Figure S4B; Table S4). The PCA and specific genes analyses showed that the PE stage of proembryo morphogenesis was unique.

We further analyzed the dynamic transcriptomes at seven different developmental stages (Figure S4; Table S5). Those genes were functionally annotated by GO enrichment analysis (Figure S4A). Cluster 12 contained genes that showed high expression at the PE stage specifically and then declined rapidly. Genes in this cluster were predominantly enriched in translation, nucleosome assembly, and photosynthesis based on GO analysis (Figure S4B). Genes in cluster 7 were expressed mainly in the GE stage and enriched in the regulation of translation and response to hormones (Figure S4C). Genes in cluster 3 maintained a high expression level in the HE stage, which enriched the regulation of cell shape, the oxylipin biosynthetic process, and the cellular amino acid metabolic process (Figure S4D). Genes in cluster 4 were expressed mainly in the TE stage, which enriched in cellular phosphate ion homeostasis and the glycerophospholipid catabolic process (Figure S4E). Cluster 11 contained genes highly expressed in the CE stage, which enriched in nutrient reservoir activity (Figure S4F). During the HE-TE-CE stage, the seeds began to accumulate nutrients and expanded rapidly, and the pods were fuller than before. Cluster 8 contained genes highly expressed in the NE stage, which enriched in autophagy and carbohydrate transport (Figure S4G). Cluster 6 contained genes that had a high expression level in the ME stage, which enriched in regulation of the cell cycle and gluconeogenesis (Figure S4H).

### 2.6. Spatial and Temporal Expression Pattern of Transcription Factors

**Figure 4:**
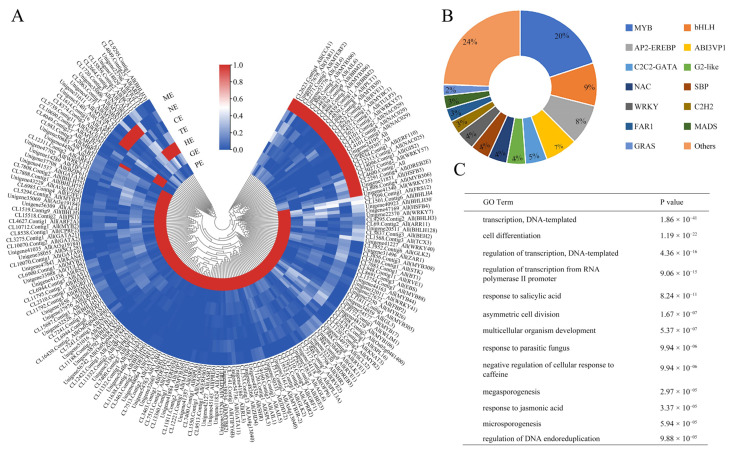
Identification of differentially expressed transcription factors (TFs). (**A**) Heatmap expression profile of the identified TFs. (**B**) Pie charts of the TFs during embryo development. (**C**) GO term analysis of TFs.

Studies have shown that transcription factors (TFs) play a vital role in regulating gene expression and participate in various important cellular processes, including hormone response, enzyme activity, development, and environmental adaptability. In this study, 176 TFs had differential expression levels during seed development, which appeared to be candidate genes for embryo-specific and mainly occurred in early embryo development (Figure 4A; Table S6).

Further, 176 TFs specifically expressed during embryo development were analyzed. 129 TFs belonging to 62 families were expressed only in the PE stage, and 4, 6, 1, 7, and 29 TFs showed specificity during the embryo developmental stages in GE, HE, CE, NE, and ME stages, respectively (Table S6). The significantly enriched TFs mainly belonged to the MYB (20%), bHLH (9%), AP2-EREBP (8%), and ABISVPI (7%) families (Figure 4B).

GO analysis showed that these differentially expressed TFs were enriched in transcription, DNA template, cell differentiation of cellular processes, regulation of the transcription of biological regulation, and the response of salicylic acid and jasmonic acid to stimuli (Figure 4C).

### 2.7. Expression Profiles of Key TFs Associated with Somatic Embryogenesis during Embryo Development of P. ostii

**Figure 5:**
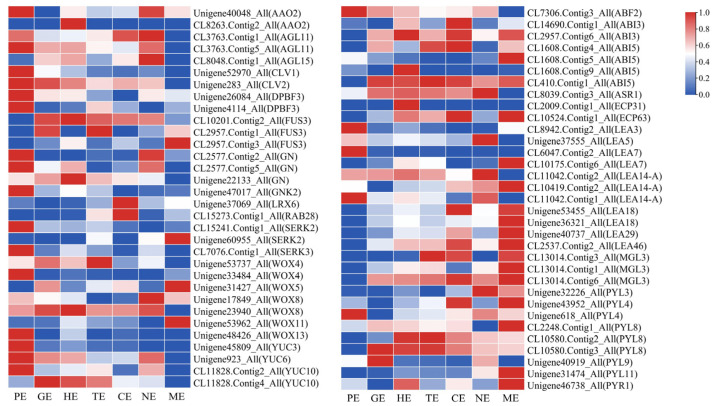
Heatmap analysis of embryo development genes in embryo development of *P. ostii*.

Many TFs play a vital role in the development of embryos. We identified many decisive TFs that were annotated to somatic embryogenesis. These TFs showed different expression patterns during embryo development (Table S7; Figure 5). *AGL11*, *AGL15*, *CLV1*, *CLV2*, GN, *SERK2*, *SERK3*, *WOX4*, *WOX8*, *WOX13*, *YUC3*, *YUC6*, *ABF2*, and *PYL4* were highly expressed in the PE stage, and they might be involved in the induction and morphogenesis of proembryo. *PYL8*, *ABI3*, *ABI5*, *FUS3*, *WOX4*, *WOX8*, *YUC10*, and *YUC6* had high expression during GE-HE-TE-CE and might involve in the division and differentiation of embryos in *P. ostii*. *ABI3*, *ABI5*, *BAK1*, *BBM1*, *BBM2*, *FUS3*, *PYL3*, *PYL4*, *PYL8*, *PYL11*, *WOX5*, *WOX8*, and *WOX11* had significant expression level in NE and ME stages, which might involve in the maturation of *P. ostii* embryo.

### 2.8. Analysis of Antioxidant Enzyme-Related Genes in Embryo Development

**Figure 6:**
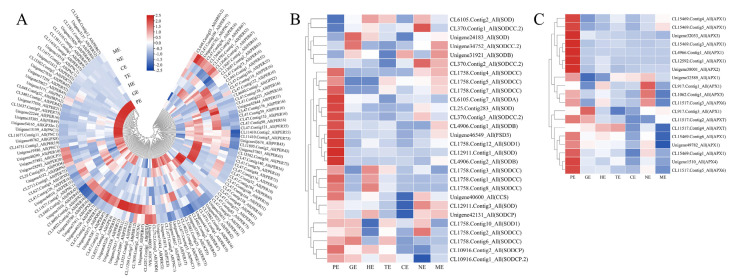
Heatmap analysis of antioxidant enzyme genes involved in embryonic development. (**A**–**C**): Heatmap of the expression of genes related to POD, SOD and APX during peony embryo development.

Antioxidant enzymes have been used effectively as efficient biochemical markers to study embryonic development. In total, 163 DEGs related to antioxidant enzymes were detected (Table S8). Among these genes, 117 DEGs, including *PER42*, *PER55*, and *PNC1* related to POD, were observed. More than half of the genes were highly expressed in the PE stage; meanwhile, POD enzyme activity is highest in the early stages (Figure 6A). 28 DEGs, including *SODCC*, *SODA*, *FSD3*, and *SOD1* of SOD, were screened out, and almost all of these genes were highly expressed in the PE stage, the SOD activity was consistent with gene expression trend (Figure 6B). Nineteen DEGs, including *APX1*, *APX3*, *APX4,* and *APX6,* related to APX were identified, and the expression trends of these genes were in good agreement with the APX activity (Figure 6C). In short, the enzymatic activity and the expression of antioxidant enzyme-related genes both had high levels in the PE stage of *P. ostii*.

### 2.9. DEGs Analysis Involved in Plant Hormones Signal Transduction and Biosynthesis during Embryo Development

**Figure 7:**
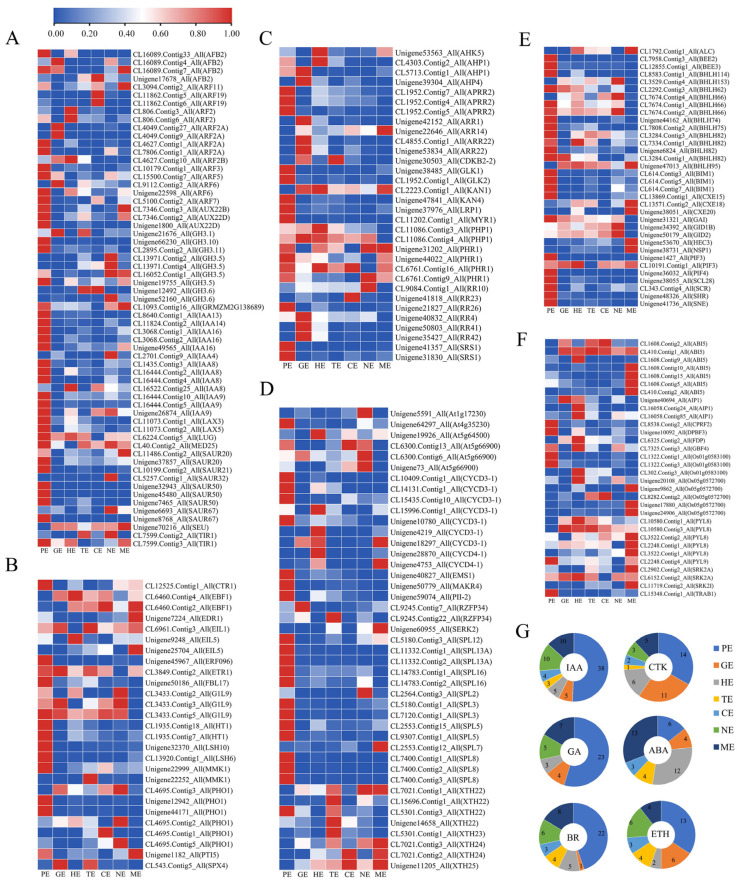
Heatmap expression profile of differentially expressed genes involved in plant hormone signaling during each embryogenesis phase. Plant-related genes of IAA (**A**), ETH (**B**), CTK (**C**), BR (**D**), GA (**E**), and ABA (**F**). (**G**), Pie charts of the DEGs involved in plant hormone signaling during embryo development.

Previous results indicated that plant hormones, especially IAA, GA, CTK and ABA, played essential roles in the different embryo development stages. The expression pattern of these plant hormones-related genes was determined in *P. ostii* (Figure 7; Table S9). The proembryo is the period of morphological construction of the embryo and is crucial for subsequent embryo formation. Therefore, we focused on the expression of various hormone-related genes during this period (Figure 5; Table S9). In total, 61 unigenes related to IAA signaling were observed; among them, 38 unigenes, including *IAA8*, *IAA13*, *ARF7*, and *SAUR50,* had a high level of expression in the PE stage and were the key to proembryo formation (Figure 7A,G). Twenty-seven DEGs related to ETH were observed, in which 13 unigenes, including *ERF096*, *LSH6*, *LSH10*, and *PHO1*, had high levels of expression in the PE stage (Figure 7B,G). Thirty-two DEGs related to CTK were observed; among them, 14 unigenes, including *APRR2*, *GLK1*, *CLK2*, *AHP4*, *ARR22*, and *SRS1*, were highly expressed in the PE stages (Figure 7C,G). Forthy-three DEGs related to BR were observed; among them, 22 unigenes, including *CYCD3*-1, *MAKP4*, *SPL3*, *SPL5*, and *SPL13A*, were highly expressed in the PE stage (Figure 7D,G). Thirty-four DEGs related to GA were observed, and 23 unigenes, including *GAI*, *PIF4*, and *SHRi*, were highly expressed in the PE stage (Figure 7E,G). The ABA genes mainly played a role in the mature period; thus, 32 DEGs related to ABA were observed. Thirteen unigenes, including *ABI5*, *PYL8*, and *SRK2A,* had high expression levels in the ME stage (Figure 7F,G). It can be seen that various hormones played an important role in embryo development, especially IAA was particularly prominent during the PE stage. This result provided a basis for the study of gene regulatory networks in early embryo development in *P. ostii*.

### 2.10. Expression Patterns of Endosperm-Related Genes during Different Embryo Development Stages

**Figure 8:**
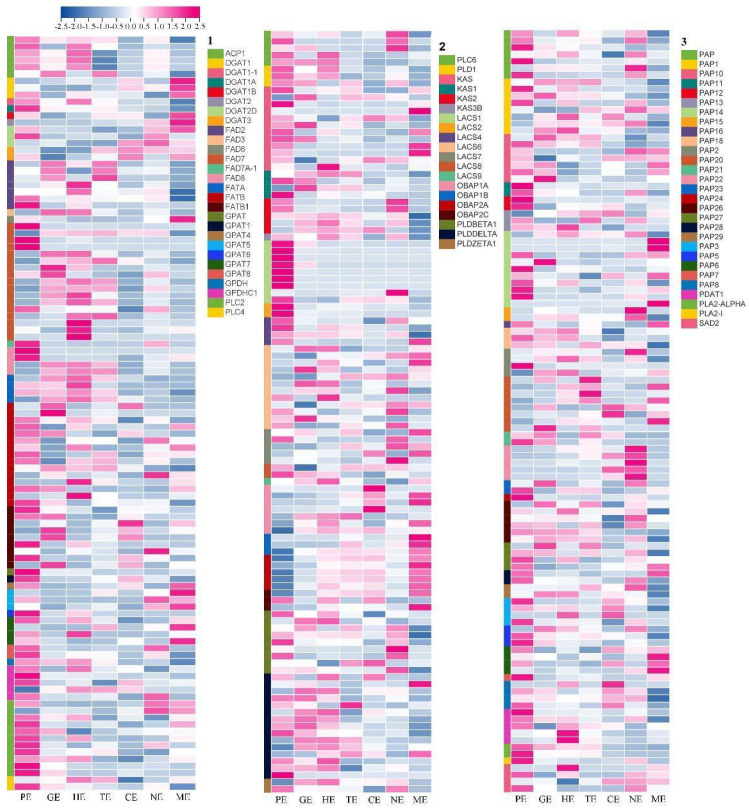
The heatmap of endosperm-related genes to lipid accumulation during *P. ostii* embryo development. 1. The genes including *ACP1*, *DGAT1*, *DGAT1−1*, *DGAT1−A*, *DGAT1−B*, *DGAT2*, *DGAT2D*, *DGAT3*, *FAD2*, *FAD3*, *FAD6*, *FAD7*, *FAD7A−1*, *FAD8*, *FATA*, *FATB*, *FATB1*, *GPAT*, *GPAT1*, *GPAT4*, *GPAT5*, *GPAT6*, *GPAT7*, *GPAT8*, *GPDH*, *GPDHC1*, *PLC2*, and *PLC4*; 2. The genes including *PLC6*, *PLD1*, *KAS*, *KAS1*, *KAS2*, *KAS3B*, *LACS1*, *LACS2*, *LACS4*, *LACS6*, *LACS7*, *LACS8*, *LACS9*, *OBAP1A*, *OBAP1B*, *OBAP2A*, *OBAP2C*, *PLDBETA1*, *PLDDELTA*, and *PLDZETA1*; 3. The genes in-cluding *PAP*, *PAP10*, *PAP11*, *PAP12*, *PAP13*, *PAP14*, *PAP15*, *PAP16*, *PAP18*, *PAP2*, *PAP20*, *PAP21*, *PAP22*, *PAP23*, *PAP24*, *PAP26*, *PAP27*, *PAP28*, *PAP29*, *PAP3*, *PAP5*, *PAP6*, *PAP7*, *PAP8*, *PDAT1*, *PLA2*−*ALPHA*, *PLA2*−1, and *SAD*.

The tree peony is an emerging oil crop, and the synthesis of unsaturated fatty acids is an important process of seed development. In this study, 328 unigenes were identified that might involve in fatty acid and triacylglycerol biosynthesis (Table S10; Figure 8). Most of these genes had high transcript levels in the GE, HE, and TE stages at 60–75 days; at this time, the morphological observation results showed that the pod expanded rapidly at this stage (Figure S1). *SAD2*, *FAD7*, *FAD6*, and *FAD8* were highly expressed in the PE stage. Among the 328 unigenes, 18 unigenes encoding oil-body oleosins showed high expression levels, specifically in the mature period. Thirty-nine unigenes encoding fatty acid desaturase were detected, including four unigenes for SAD, eight unigenes for oleate desaturase (seven for *FAD2* and one for *FAD6*), and 23 unigenes for omega-3 fatty acid desaturase (one for FAD3, 18 for *FAD7* and four for *FAD8*). In particular, *SAD2*, *FAD2*, *FAD3*, *FAD7*, and *FAD8*, which encoded fatty acid desaturase with high expression levels in the fast oil accumulation stage, were identified in *P. ostii*.

### 2.11. Prediction of the Protein-Protein Interaction Network in the Proembryo Development of Tree Peony

**Figure 9:**
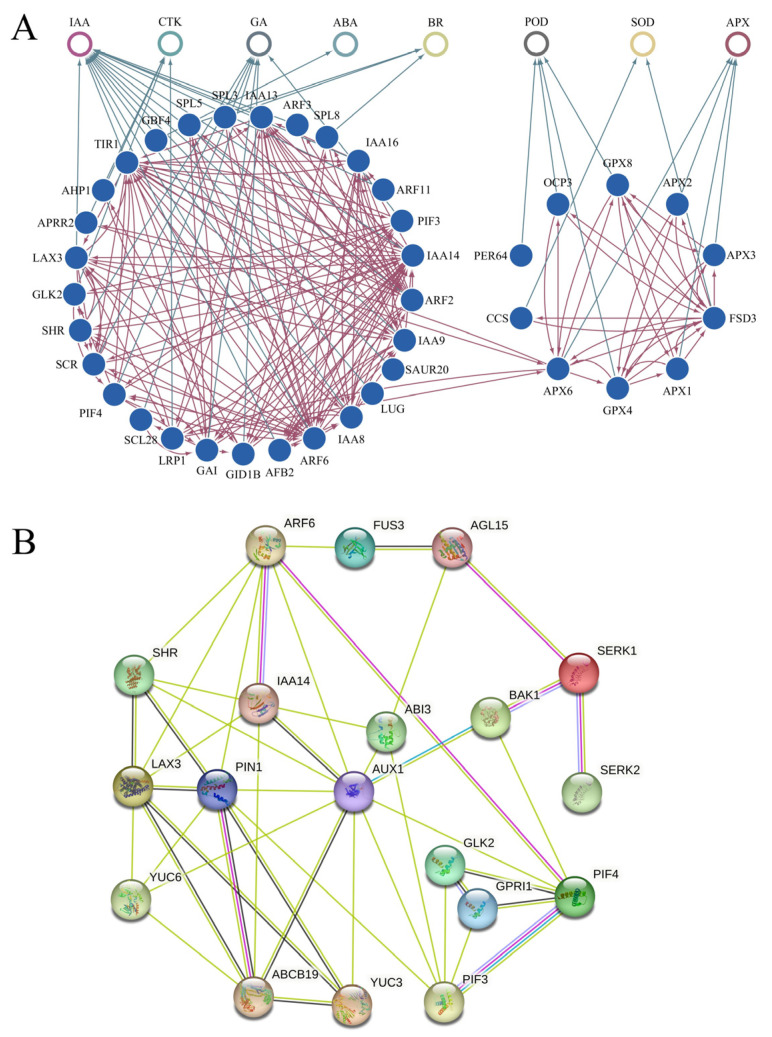
Network interaction prediction map of some key genes involved in the early embryo development of *P. ostii*. (**A**) The primary PPI network in the early embryo development of *P. ostii*; (**B**) The core PPI network with 19 key proteins in the early embryo development of *P. ostii*.

The morphogenesis of early embryos was crucial for embryo development. This study showed that numerous genes related to IAA, GA, BR, CTK, ABA, and enzyme were actively expressed in the PE stage and participated in the early morphogenesis of the embryos in *P. ostii*. We tried to construct a protein interaction network (PPI) with IAA as the center and multiple hormones and enzyme activities involved in early embryo development (Figure 9A). A more refined PPI for 19 key proteins including GLK1, GLK2, PIF3, PIF4, AUX1, PIN1, LAX3, SHR, BAK1, SERK1, SERK2, IAA14, AGL15, ABI3, ABCB19, YUC3, YUC6, ARF6, and FUS3 were predicted (Figure 9B). Six central node proteins, including AUX1, PIN1, ARF6, LAX3, ABCB19, PIF3, and PIF4, were screened, which could be of great value in early embryo development.

### 2.12. The Validation of RNA-Seq Data by qRT-PCR

**Figure 10:**
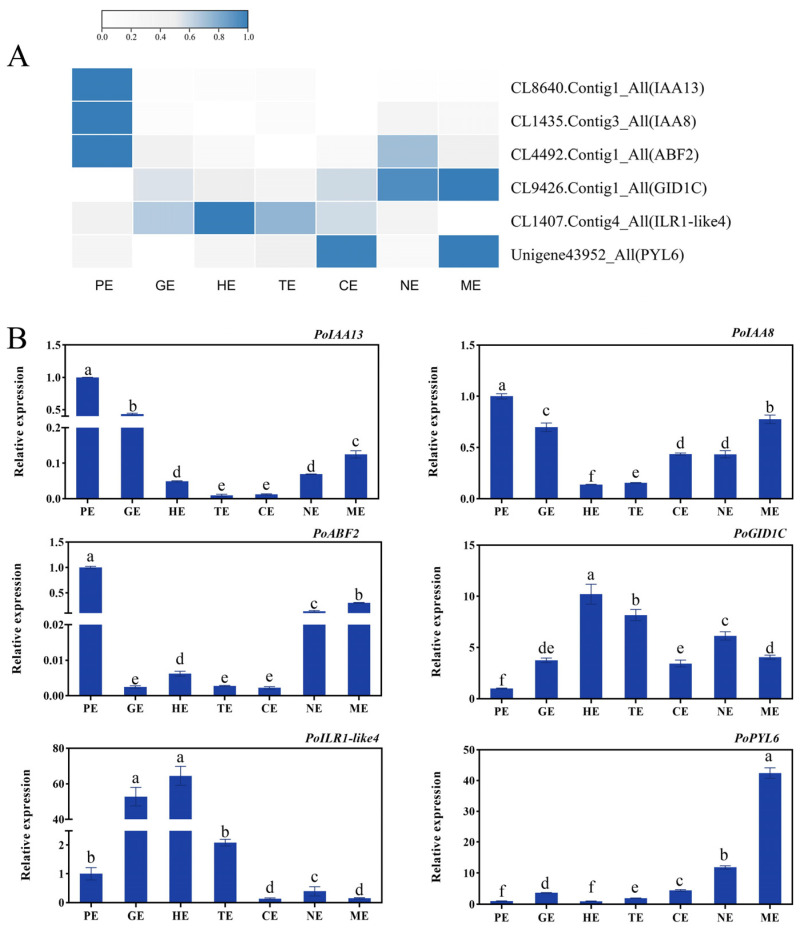
The validation of RNA-Seq data. (**A**) Heatmap expression profile of the embryo development-related genes based on RNA-seq. (**B**) Validation of transcriptome data by qRT-PCR. The letters above the bars indicates the significance among different samples. Bars represent means ± standard error (n = 3). *p* < 0.05, one-way ANOVA.

To verify the accuracy of the gene expression patterns, we selected several genes for qRT-PCR validation. The results showed that the IAA-related genes of *IAA3*, *IAA8* and ABA-related *ABF2* expressed at the highest level during the PE stage; they were involved in the morphogenesis of the embryo. The IAA-associated *ILR1*-*like4* gene was highly expressed during embryonic differentiation. The gibberellin-associated *GID1C* and ABA-associated *PYL6* maintained high levels of expression during middle and late embryonic development, and they may be involved in the maturation of the embryo. The expression patterns determined by qRT-PCR were consistent with the overall trend results of the transcriptome (Figure 10A,B), which indicates that the gene expression profiles of the transcriptome were convincing.

## 3. Discussion

**Figure 11:**
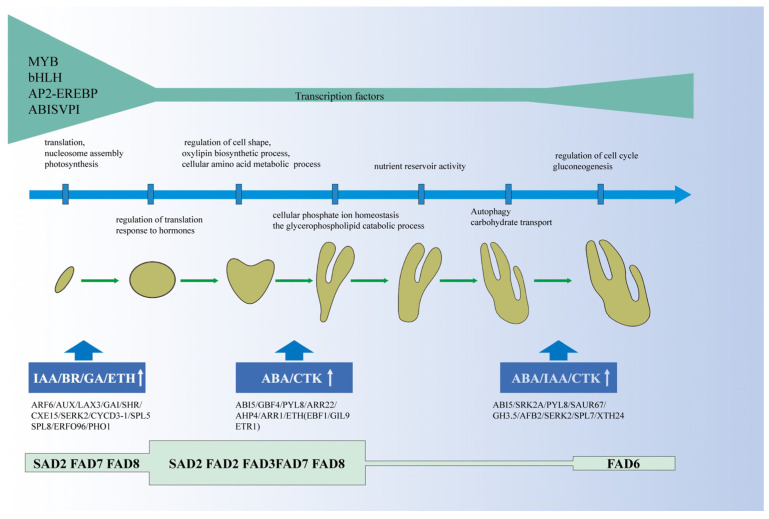
Working model of embryo development in *P. ostii*.

It is of great significance to clarify the key period of embryo development and the spatiotemporal expression profile of genes for embryonic development and even somatic embryo development research. In this study, the morphological and anatomical observations of the process of embryo development from pollination to embryo maturation were detailed, and the gene expression profile of plant hormones, enzyme activity-related genes, and transcription factors during embryo development was detected by RNA-seq technology in *P. ostii*. Finally, we proposed a working model to illustrate the gene expression modules and possible molecular mechanisms underlying embryo development in *P. ostii* (Figure 11).

### 3.1. Growth and Development of P. ostii Seed

For species of sexually reproducing plants, embryonic development is a crucial reproductive stage; the fertilized egg is a totipotent cell that establishes the main cell and tissue lineages of adult plants through cell division and cell identity specification during early embryogenesis [55]. The fertilized egg continued to divide into two cells, four cells, eight cells, and 16 cells and became a globular embryo when it was divided into 32 cells. Maturation stages include heart, torpedo, cotyledon, mature green, and dry seed [55]. Similar to *Arabidopsis*, the morphological transition state of peony embryo also experienced proembryo, globular, heart, torpedo, cotyledon, and maturation, which was consistent with the classical embryonic development process of dicotyledonous plants. The zygotic embryo takes 40 days to maturity from fertilization and is a globular-heart embryo at 5 days post-pollination in *Capsicum chinense* Jacq [56]. The zygotic embryo became mature at 28 days after pollination, while it formed into a globular embryo at 5 days post-pollination in *Medicago truncatula* [57]. We observed that the zygotic embryo took 130 days to mature, but it was still a globular embryo at 60 days post-pollination; the morphological establishment of the embryo takes a long time, while a large number of genes were significantly expressed during this time (Figure S3B). This shows that embryo morphogenesis is an extraordinarily complex process in *P. ostii*; it is recommended that researchers carry out more detailed time-point gene expression studies in the future.

In this study, the development of seeds was divided into seven stages according to the developmental state of the seed embryo in *P. ostii*. The researchers collected *P. ostii* seeds in Yangzhou (China) and found that globular, heart, and torpedo embryos were formed 45, 55, and 60 days after pollination [58], while we collected materials in Zhengzhou (China) and found that globular, heart, and torpedo embryos formed 60, 65, and 75 days after pollination, respectively; this difference may be related to the planting location of *P. ostii* and the environmental temperature. However, it has been reported that 55–85 days is a period of rapid fatty acid biosynthesis, which may provide sufficient nutrient reserves for the transition from torpedo embryos to cotyledon embryos [46,59]. We found that at 60–75 days (GE-HE-TE), related genes of FAD and SAD were highly expressed, and the pods rapidly expanded (Figure 1 and Figure 8). It can be seen that the endosperm developed rapidly during this period, which provided sufficient nutritional support for the morphological transformation of the embryos.

### 3.2. Genes Related to Plant Hormones and Enzymes Were Important for the Embryo Development of Tree Peony

Auxin plays an important and decisive role in early embryonic morphogenesis [60]. In this study, we found that numerous auxin-related genes were highly expressed in the early stage of *P. ostii* embryo development (Figure 7). A previous study showed that auxin synthesized by YUC flavin monooxygenase was a key source of auxin for embryogenesis, and *YUC1*, *YUC4*, *YUC10*, and *YUC11*, involving in IAA biosynthesis redundantly regulate embryogenesis and post-embryonic organ formation [24]. Our study also found the expressions of IAA synthesis genes *YUCC3*, *YUCC6*, *YUCC10*, and *AAO2* were specifically up-regulated in the proembryo of *P. ostii*, and it can be seen that those genes involved in the synthesis of IAA in early embryogenesis (Figure 7). *AUX1*, *LAX1*, and *LAX2,* which regulate IAA transport, were highly expressed in the early embryo development of *Arabidopsis* [22,61]. In *P. mongolica*, *AUX1* and *PIN3* were also highly expressed in early embryogenesis [41]. Our study found *LAX3* and *LAX5* specifically highly expressed and regulated IAA transport at the proembryo of *P. ostii*. In addition, *SAUR20*, *SAUR21*, *SAUR50*, *SAUR67*, *GH3*.5, *GH3*.10, GH3.11, *ARF2A*, *ARF3*, *ARF5*, and *ARF6*, related to IAA signal transduction, actively expressed in the early embryo development of *P. ostii*. In summary, the genes related to auxin synthesis, transport, and signal transduction, jointly regulated auxin content and further regulated the formation and development of proembryos in *P. ostii*.

### 3.3. The Molecular Study on Embryo Development Provided the Basis for Somatic Embryo Culture Technology in Tree Peony

The somatic embryo regeneration pathway was an important part of the plant tissue culture and rapid propagation system. The somatic embryogenesis pathway has problems such as asynchronous differentiation and further development of somatic embryos, malformations, disturbed polarity, precocious germination, early loss of embryogenic potential, and strong genotypic differences in regeneration efficiency [62]. The above-mentioned problems also existed in the somatic embryonic development of tree peonies. Many studies have shown that somatic embryos and zygotic embryos were similar in morphological characteristics, even at the biochemical level, especially the similarity of gene expression products and protein components [53,54,62]. Therefore, the molecular mechanism of seed embryo development can provide a theoretical and technical basis for the development of somatic embryos of tree peonies.

*WOX* homeodomain transcription factors regulate early embryo patterns. *WOX8* was involved in the regulation of zygote polarization in *Arabidopsis* [63], while *WOX2*, *WOX8*, and *WOX9* were important early pre-embryonic cell fate regulators [64,65]. *VvWOX2* and *VvWOX9* were early expression markers of SE development in Vitis vinifera [66]. In *P. ostii* SE, *WRKY2*, *WOX9*, and *WOX11* were highly expressed in zygotic embryo explants [6]. In this study, *WOX4*, *WOX8*, and *WOX13* were all highly expressed in PE, and they promoted embryonic morphogenesis in the zygotic embryo. *WOX4* and *WOX8* also had high expression levels in GE, HE, and TE, while *WOX5* and *WOX11* were highly expressed in ME. *SERK* was reported to be positively regulated during zygotic embryogenesis and somatic embryogenesis, and it was a key factor regulating embryogenesis [67,68,69]. In this study, *SERK2* and *SERK3* genes were detected during zygotic embryo development and had high expression in the PE stage, while *SERK2* was also highly expressed in the ME period. *SERK2* was involved in the morphogenesis of the early stage and the embryonic maturation of the late stage in *P. ostii*. *LEC1*, *ABI3*, *FUS3*, and *LEC2* are called LAFL factors, and they are necessary and sufficient for embryo development and regulate various development processes [10,11]. In this study, *FUS3* was highly expressed during GE-HE-TE-CE, and *ABI3* had significant expression levels in CE and ME stages. However, we didn’t find *LEC1* and *LEC2* genes, which may play a specific role in earlier embryo development.

In this study, we predicted the complex network of protein interactions formed in early embryos based on gene expression profiles (Figure 9). Studies in *Arabidopsis* showed that ARF6 has protein interactions with IAA14, PIN1 has protein interactions with ABCB19, SERK1 have protein interactions with AGL15, and BAK1 [70,71,72]. Genes, including SERK2, FUS3, and AGL15, played an important role in the development of somatic embryos in tree peonies [6], but the interaction among these proteins has not been fully verified in tree peonies. In order to better study somatic embryos, the PPI network could be used as the basis for further exploration in tree peonies.

In summary, we successfully constructed the gene expression profile of seed development and predicted the PPI of early embryo development. Therefore, the research on the molecular regulation mechanism of *P. ostii* embryo development may solve the problem of somatic embryo regeneration and provide a new approach and molecular biology theory and technical support for tree peonies.

## 4. Materials and Methods

### 4.1. Plant Material

Seeds of *P. ostii* were collected from Henan Agricultural University, China. The plants had been grown under the same environmental and cultivation conditions for 10 years. When the male parent flower bud was in the loose bud stage, it was transported to the laboratory. Sterile tweezers were used to remove the anthers and place them in a sterile petri dish and then in a dry, ventilated location without direct sunlight for 24 h, after which they were stored in a refrigerator at 4 °C for later use. When the female parent flower bud was in the loose bud stage, tweezers were used to remove the stamens and place them in a paper bag with sulfuric acid. Then, 2–3 days after emasculation, pollination was performed when the mucus secreted by the stigma of the pistil was bright in color. Pollination was carried out before 10 a.m. as follows: the dried pollen was dipped onto the stigma of the female parent with a brush, and the paper bag was covered immediately after pollination. When the stigma wilted after 10 days, and there was no possibility of fertilization, the bag was removed to avoid hindering the growth of the fruit.

We observed the seed development process from pollination until maturation from 1 April to 15 August 2018. Pods were hand-collected at intervals of 5 days, from the beginning of podding until full maturity, covering a total of 130 d. The pods are collected, and removed the seed coat and endosperm as much as possible, retaining the embryonic part. Paraffin-embedded samples were fixed in FAA (3.7% formaldehyde, 5% glacial acetic acid and 50% ethanol) and stored at room temperature to observe the developmental stages. For transcriptome and qRT-PCR sampling, the sample was quickly cooled in liquid nitrogen and stored in a refrigerator at −80 °C for later use.

### 4.2. Histology Observation

According to the previously described method of Zhang et al.’s study [73], the fixed samples were stained with safranin O for 12–15 h and dehydrated in a graded series of ethanol (70%, 85%, 95% and 100%), followed by a xylene/ethanol series (xylene/ethanol 1:3, 1:1, 3:1 and 100% xylene). Xylene was replaced gradually with paraffin (Paraplast Plus, Sigma, Chemical Co. St. Louis, MO, USA, P3683) at 60 °C for 2 days with four replacement events of paraffin; then, 20-μm sections were made using a microtome and were stained with Fast Green Stain for 20 s. Safranin O stains the cell nucleus, while fast green stains the cell wall.

### 4.3. Measurement of Physiological Data

The pods were collected, and the seed coat was removed on days 5, 60, 65, 75, 90, 110, and 130 of seed development, removing most of the endosperm as possible and retaining the embryo part. The antioxidant enzyme activity of SOD, POD, APX, and CAT was measured [74,75]. Three biological replicates were included for each treatment, and three technical replicates were included for each biological replicate.

### 4.4. Total RNA Extraction and RNA-Seq Library Preparation

For transcriptome sampling, seven key stages of embryo development (5, 60, 65, 75, 90, 110 and 130) were determined based on the results of the paraffin section analysis. Total RNA was extracted from the embryos using a Quick RNA extraction Kit (Huayueyang, Beijing, China), and RNA was converted to cDNA using a kit with gDNA Clean (AG, Changsha, China) according to the manufacturer’s instructions. Subsequently, total RNA was qualified and quantified using a NanoDrop and Agilent 2100 bioanalyzer (Thermo Fisher Scientific, Waltham, MA, USA).

Purified mRNA was obtained by enrichment of the oligo-dT magnetic bead method; then reverse transcribed to synthesize cDNA. Data filtering using SOAPnuke1.4.0 software and alignment of clean reads to reference gene sequences by Trinity. Transcript abundance was determined by FPKM values. Principal component analysis (PCA) was performed using the prcomp function in R software (accessed on 14 March 2019) [76].

### 4.5. Transcriptome Analysis and Genes Expression Patterns Analysis

The unigenes were annotated by functional databases (NT (ftp://ftp.ncbi.nlm.nih.gov/blast/db/, accessed on 14 March 2019), NR (ftp://ftp.ncbi.nlm.nih.gov/blast/db/, accessed on 14 March 2019), SwissProt (https://www.uniprot.org/, accessed on 14 March 2019), KEGG (http://www.genome.jp/kegg/, accessed on 14 March 2019), GO (http://geneontology.org/, accessed on 14 March 2019), Pfam (http://pfam.xfam.org/, accessed on 14 March 2019), and KOG (https://www.ncbi.nlm.nih.gov/COG/, accessed on 14 March 2019)).

GO enrichment analysis and KEGG enrichment analysis were performed using Phyper, a function of R packages (accessed on 14 March 2019) [77]. The significance levels of terms and pathways were corrected by the Q value with a rigorous threshold (Q value < 0.05).

DEGs were screened using |log 2 FC| > 1 and *p* < 0.05 by applying DEseq2. The PE-specific genes with FPKM < 1 in all embryo samples and FPKM ≥ 3 in any of the PE samples, and so on.

### 4.6. The Prediction of Protein-Protein Interaction Network

The DEGs related to hormone, enzyme, and transcription factors that related to SE that were highly expressed in the proembryo stage were screened to construct a comprehensive primary interaction network, and these DEGs were compared with STRING v11.5 (https://string-db.org/, accessed on 4 November 2022) [78] database to obtain the primary protein-protein interaction network of them, Cytoscape3.3.0 software was used for visualization. In addition, in order to explore the interaction network between the key genes of somatic embryogenesis and the related genes of hormones and antioxidant enzymes in the early embryonic period, 19 key proteins, including 14 proteins with strong interactions ability from the primary PPI network and five key somatic embryogenesis proteins were screened to construct a secondary protein-protein interaction network.

### 4.7. Validation of Transcriptome Data by qRT-PCR

The material for qRT-PCR and transcriptome sequencing was consistent. Extraction of total RNA and reverse transcription of cDNA were performed, and gene expression levels were calculated using the 2^−ΔΔCt^ method, with *PsActin* as the endogenous reference gene [79]. The primer list is shown in Table S1.

## 5. Conclusions

Tree peony integrates ornamental, medicinal, and oil values and has become an emerging oil crop. The detailed analysis of its embryo development process has important economic and scientific value. This study found that the process from proembryo to heart embryo lasted for 60 days, followed by rapid embryo division and differentiation, while the pod became full. *SAD2*, *FAD2*, *FAD3*, *FAD7*, and *FAD8,* which encoded fatty acid desaturase had high expression levels in the fast oil accumulation stage in *P. ostii*. The antioxidant enzyme activity of seeds was particularly active in the early stages but decreased and remained stable in the later stages; the expression of enzyme-related genes also showed a similar trend. Various related genes of IAA, GA, BR and ETH were specifically overexpressed during proembryo formation, while CTK and ABA may be the main regulators of embryo morphogenesis and maturation. We predicted a protein-protein interaction network of key proteins during early embryo development, which is centered on AUX1-PIN1-ARF6-LAX3-ABCB19-PIF3-PIF4. This study provides new insights into the formation of embryo development and even somatic embryo development of *P. ostii*.

## Data Availability

The data that support the findings of this study are openly available within this manuscript and its supporting materials. The raw data were uploaded to the BIG data center (https://bigd.big.ac.cn/, accessed on 10 May 2023) with project No. PRJCA016892.

## Supplementary Materials

The following supporting information can be downloaded at: https://www.mdpi.com/article/10.3390/ijms241310715/s1.
