# Supplementary material for: Transcriptome Landscape Analyses of the Regulatory Network for Zygotic Embryo Development in Paeonia ostii
Source: Int J Mol Sci. 2023 Jun 27;24(13):10715. doi: 10.3390/ijms241310715 (PMC10342179; doi:10.3390/ijms241310715)
Supplement: Supplementary file 1 [file ijms-24-10715-s001.zip › Captions for supplementary material list.pdf]

Captions for supplementary material:

Figure S1. Functional annotation of the transcriptome. (A-C) Distribution of the annotated BP, CC and MF in the GO database based on the detected unigenes of the *P. ostii* transcriptome of developing seeds; (D) Eukaryotic orthologous groups (KOG) classification of assembled unigenes.
Figure S2. Global analysis of transcriptome in *P. ostii* embryo development. (A) The up- and down-regulated genes based on data from the six groups. (B) Species distribution of the top BLAST hits of tree peony sequences with other plant species.
Figure S3. The repeatability and similarity analysis of all samples and gene expression analysis in 7 stages of *P. ostii* embryo development. (A). Principle component analysis (PCA) of the RNA-seq datasets. (B) The relative expression pattern of all specific expressed genes.
Figure S4. Co-expression analysis of differentially expressed genes. (A) The DEGs expression cluster of embryo development in *P. ostii*; (B-H). The co-expression pattern (left) and the enriched GO terms in biological processes (right) of cluster PE (B), GE (c), HE (D), TE (E), CE (F), NE (G), and ME (H). The red lines show the normalized FPKM of individual genes.
Table S1. Primers used for qRT-PCR in this study.
Table S2. Summary of RNA-Seq data and reads of the transcriptome.
Table S3. The up and down genes of the transcriptome.
Table S4. All-specific expression genes of the transcriptome.
Table S5. Cluster genes of embryo development in this research.
Table S6. TFs of seed development in this research.
Table S7. Enzymes or proteins related to lipid accumulation of embryo development in *P. ostii*.
Table S8. DEGs related to plant hormones during embryo development in *P. ostii*.
Table S9. DEGs related to antioxidant enzyme during embryo development in *P. ostii*.
Table S10. Genes for Embryogenesis during embryo development in *P. ostii*.
