# Supplementary material for: Transcriptome Landscape Analyses of the Regulatory Network for Zygotic Embryo Development in Paeonia ostii
Source: Int J Mol Sci. 2023 Jun 27;24(13):10715. doi: 10.3390/ijms241310715 (PMC10342179; doi:10.3390/ijms241310715)
Supplement: Supplementary file 1 [file ijms-24-10715-s001.zip › ijms-2420130-Figure S1-S4.pdf]

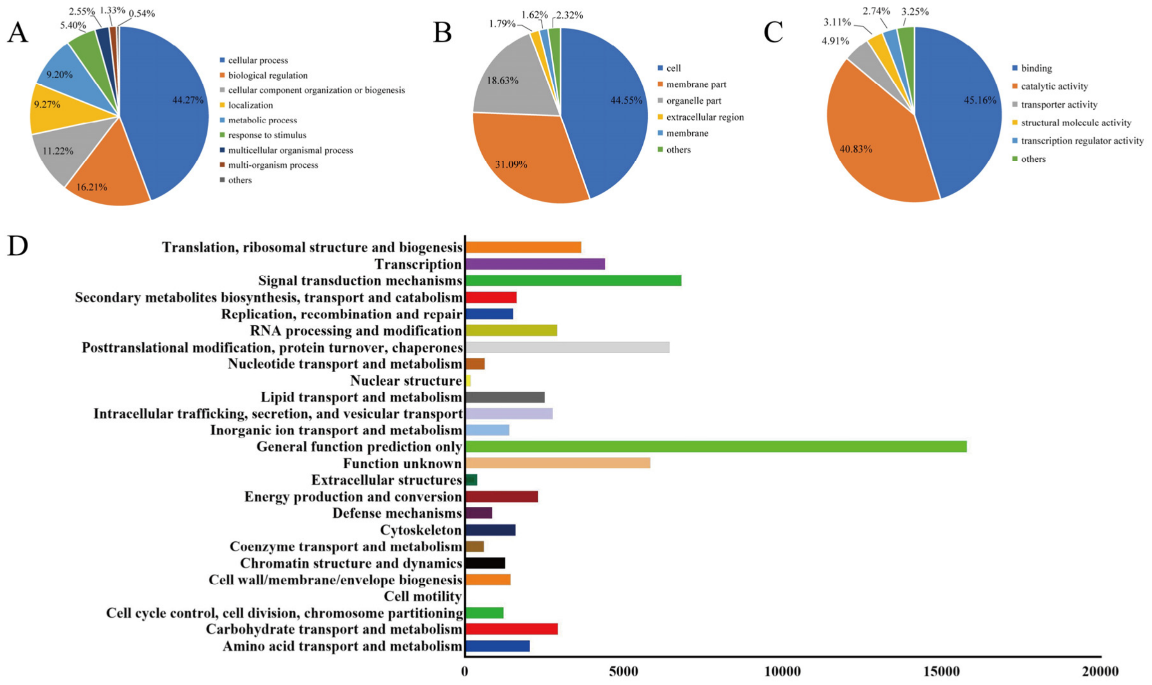

Figure S1. Functional annotation of the transcriptome. (A-C) Distribution of the annotated BP, CC and MF in the GO database based on the detected unigenes of the *P. ostii* transcriptome of developing seeds; (D) Eukaryotic orthologous groups (KOG) classification of assembled unigenes.

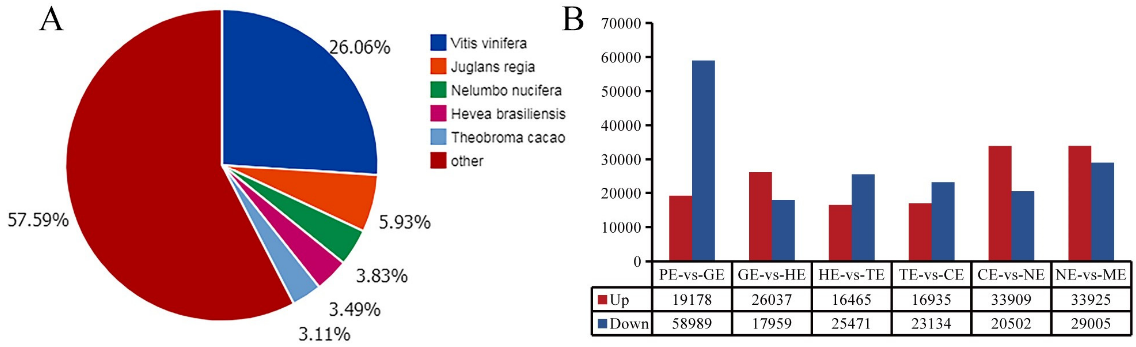

Figure S2. Global analysis of transcriptome in *P. ostii* embryo development. (A) The up- and down-regulated genes based on data from the six groups. (B) Species distribution of the top BLAST hits of tree peony sequences with other plant species.

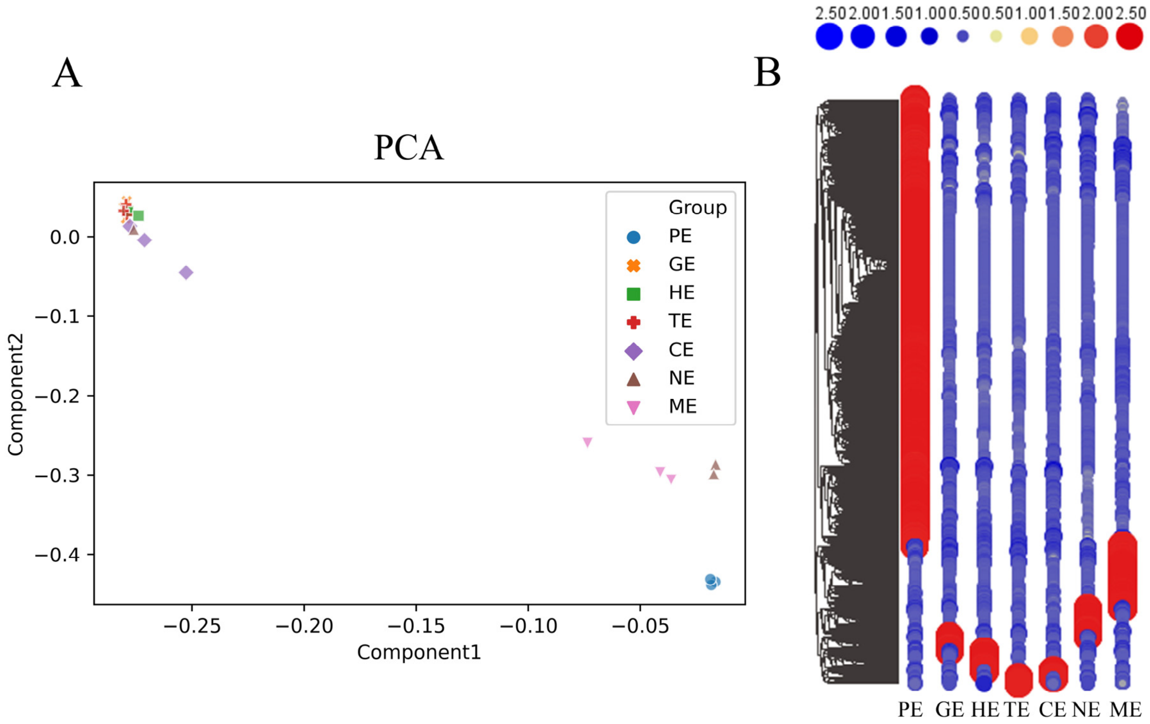

Figure S3. The repeatability and similarity analysis of all samples and gene expression analysis in 7 stages of *P. ostii* embryo development. (A). Principle component analysis (PCA) of the RNA-seq datasets. (B) The relative expression pattern of all specific expressed genes.

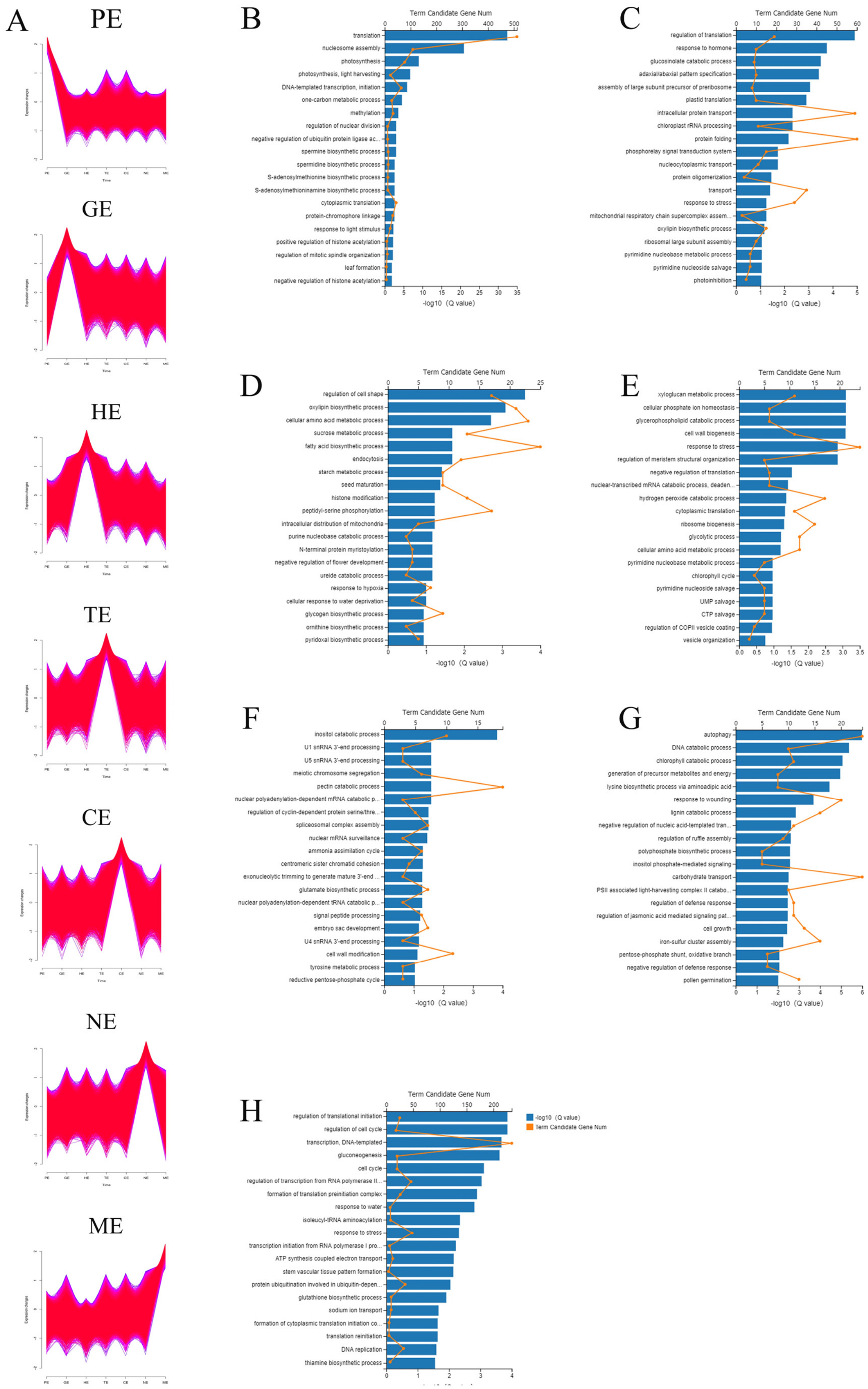

Figure S4. Co-expression analysis of differentially expressed genes. (A) The DEGs expression cluster of embryo development in *P. ostii*; (B-H). The co-expression pattern (left) and the enriched GO terms in biological processes (right) of cluster PE (B), GE (c), HE (D), TE (E), CE (F), NE (G), and ME (H). The red lines show the normalized FPKM of individual genes.
